# Assessment of Pressure Sources and Water Body Resilience: An Integrated Approach for Action Planning in a Polluted River Basin

**DOI:** 10.3390/ijerph15020390

**Published:** 2018-02-23

**Authors:** Domenica Mirauda, Marco Ostoich

**Affiliations:** 1 School of Engineering, Basilicata University, Viale dell’Ateneo Lucano 10, 85100 Potenza, Italy; 2 Veneto Regional Environmental Prevention and Protection Agency (ARPAV), Provincial Department of Venice, Via Lissa 6, 30172 Venice-Mestre, Italy; marco.ostoich@arpa.veneto.it

**Keywords:** water quality, integrity model, pollution load, resilience, decision-support system, river basin, ecological macro-descriptors, pressure sources, Water Framework Directive, wastewater-treatment plant

## Abstract

The present study develops an integrated methodology combining the results of the water-quality classification, according to the Water Framework Directive 2000/60/EC—WFD, with those of a mathematical integrity model. It is able to analyse the potential anthropogenic impacts on the receiving water body and to help municipal decision-makers when selecting short/medium/long-term strategic mitigation actions to be performed in a territory. Among the most important causes of water-quality degradation in a river, the focus is placed on pollutants from urban wastewater. In particular, the proposed approach evaluates the efficiency and the accurate localisation of treatment plants in a basin, as well as the capacity of its river to bear the residual pollution loads after the treatment phase. The methodology is applied to a sample catchment area, located in northern Italy, where water quality is strongly affected by high population density and by the presence of agricultural and industrial activities. Nearly 10 years of water-quality data collected through official monitoring are considered for the implementation of the system. The sample basin shows different real and potential pollution conditions, according to the resilience of the river and surroundings, together with the point and diffuse pressure sources acting on the receiving body.

## 1. Introduction

In most countries the water supply has been increasingly threatened: on the one hand, by growing water demand for civil, industrial and agricultural uses, energy production, and in the transport and tourism sectors; on the other hand, by the worsening quality of surface and groundwater bodies endangered by pollution, climate change, and hydro-morphological alterations due to human activity. For these reasons, the resource, thought to be unlimited until a few decades ago, is now facing shortages. In fact, global water demand is estimated to increase by over 40% by 2030 [1]. In addition, the status of rivers, lakes and wetlands is at risk, sometimes irreversibly.

In Europe, growing awareness about resource vulnerability and depletion, and about the urgent need to adopt an integrated water-management and safeguarding policy, led to the release of Water Framework Directive 2000/60/EC—WFD [2]. This introduced innovative elements, such as dealing with water-management (from quality control to flood protection) holistically and using a wider spatial scale at the basin or even the water-district level. The WFD also suggested analytical tools able to assess the water bodies’ vulnerability to existing pressures and, then, direct the planning of mitigation interventions against the environmental pollution risk. In view of this, the WFD leads a new integrated approach to water protection, improvement and sustainable use, and provides a coherent management framework with the aim of meeting its goals in an integrated way [3,4,5]. In order to implement this integrated and holistic approach, important laws were subsequently adopted that established more detailed regulations on specific aspects: Directive 2006/118/EC [6] on the protection of groundwater against pollution and deterioration; Directive 2006/7/EC [7] concerning the management of bathing-water quality; Directive 2007/60/EC [8] on the assessment and management of flood risks; and Directive 2013/39/EU [9] regarding chemical pollution of surface water. The complete and correct fulfillment of these requirements is, in fact, an essential condition for the achievement of the WFD objectives.

More recently, the European Commission, in its 10-year strategy for growth and employment in Europe released in 2010, highlighted the need for a new water-management policy based on a more efficient, green and competitive use of this resource, in order to overcome the current economic and environmental crisis, adapt to climate change, and increase resilience to natural catastrophes [10].

In Italy, Legislative Decree No. 152/2006 [11] fully adopted the WFD and all its innovative aspects, among which was the concept of the integrated and coordinated management of water as well as that of the vulnerability assessment of a river to pollutants. The vulnerability assessment can be performed by adopting both qualitative and quantitative approaches. The former identify how changes in human activities or climate affect water systems, proposing adaptation measures to reduce vulnerability. The latter use either indicator or function methods. Among the qualitative approaches, Vörösmarty et al. (2000) [12] used numerical experiments combining climate-model outputs, water budgets, and socio-economic information along digitised river networks. Turnes et al. (2003) [13] introduced a framework for qualitative assessment based on sensitivity, resilience and exposure. An indicator approach to climate change was discussed by Sullivan and Meigh (2005) [14], while Feng and He (2009) [15] considered water-quantity change, water security, ecological security, and management processes in analysing China’s cross-border water resources. Indicator methods are more common among the quantitative approaches. The system developed by Hamouda et al. [16] was based on the drivers–pressures–state of the environment–impacts–responses (DPSIR) model. The analytic hierarchy process and the Delphi (expert scoring) method were used by many others [17,18,19]. Bay et al. (2012), Dixon (2005) and Liu et al. (2012) [20,21,22] applied fuzzy logic to determine the most appropriate indicators and how to weight multiple indicators. Function methods [23,24], instead, consider the vulnerability of water resources as a function of sensitivity and pressure resistance under climate change and human activities. These methods are more systematic, despite some limitations such as the index selection and the requirement of greater mathematical competence for operators.

The present study proposes an integrated approach, combining the results of the water-quality classification, according to the Macro-descriptors Pollution Level (LIM) and the Macro-descriptors Pollution Level for the Ecological status (LIMeco), with those of a mathematical integrity model. This methodology was applied in the period 2007–2015 to the Bacchiglione, a sample basin in north-east Italy. In detail, the water quality of the river from 2007 to 2010 is based on knowledge of the trophic status according to the LIM [25], built on the following chemical and physical parameters collected from official monitoring: dissolved oxygen in % saturation (DO%); five-day test for biochemical oxygen demand (BOD_5_); chemical oxygen demand (COD); ammonium nitrogen (NH_4_-N); nitrate nitrogen (NO_3_-N); total phosphorus (P); and *Escherichia (E.) coli*. From 2010 to 2015, the classification was also performed through the LIMeco, introduced by ministerial decree n. 260 of 2010 [26], which replaced the LIM indicator [25]. This new indicator is defined on the basis of a lower number of parameters (DO%, NH_4_-N, NO_3_-N) compared to the LIM indicator. Total P, BOD_5_, COD, and *E. coli* are no longer included in the calculation of LIMeco (Appendix A).

The mathematical model, based on the theory of influence diagrams [27,28,29], detects the river integrity or vulnerability to the effects of point and diffuse pollution sources within the basin. In this work, the focus is identifying the potential impacts of urban wastewater, which is among the most important causes of river degradation in the basin investigated. In fact, besides numerous industrial settlements, the water quality of the Bacchiglione is strongly affected by high population density and the unrestricted presence of tourists all year round. Such an extremely anthropised area could produce a large amount of organic, nutrient, and microbiologic loads which, if not appropriately treated, affect the quality of the receiving water body. Although Directive 271/91/EEC [30] requires all the civil settlements higher than 2000 population equivalent (PE) to be served by an adequate wastewater treatment plant (WWTP) network, it does not always allow the achievement of the quality objectives for water bodies established by [2].

In this study, the integrity model was implemented according to two pressure-source indices: the Non-Treated Loads index and the Total Pollutant Reduction index. The first index takes into account both the self-depuration capacity of the river itself together with the influence of the diffused loads, untreated or eluded by the treatment plant. The second estimates how the water body is able to protect itself from anthropic loads acting on the basin. Such a model, previously developed in the same river in the period 2001–2003 [31], has now been recalibrated to a period of nearly 10 years and on a wider range of chemical and physical parameters. This allowed a more complete and exhaustive analysis of a complex system, such as a hydrographic basin, to be obtained based on a plurality of parameters, and the model accuracy over a longer period of time to be verified. Besides, the combined reading of the mathematical model results in information on basin morphology and land use that had not been discussed in the previous work [31], and better explains some forms of behaviour of the water body and its surrounding territory. In fact, recent studies [32,33] have underlined how the hydromorphological aspect of a river, linked to the aquatic habitat and ecosystem health, plays an important role in the assessment of its ecological status which, if undetected, could undermine the overall innovative approach of WFD.

The proposed integrated methodology, comparing the vulnerability or integrity scenarios depicted by the two indices and considering the water-quality classification system, represents a useful support tool for municipal decision-makers when planning and selecting correct interventions within the basin; more specifically, in the case of bad water quality, by addressing monitoring activities and detecting the best source of pollution mitigating actions; and in the case of “good” water quality, managing the territory in order to eliminate the potential causes of water-body degradation. Finally, if this methodology is implemented on an online platform, it could even help communicate the river quality status to the population in a fast and simple way, showing timely and continuous up-to-date information on various vulnerability scenarios, and consequently speeding up decision-making processes and thus real and potential interventions [34,35].

## 2. Description of the Bacchiglione Basin

**Figure 1:**
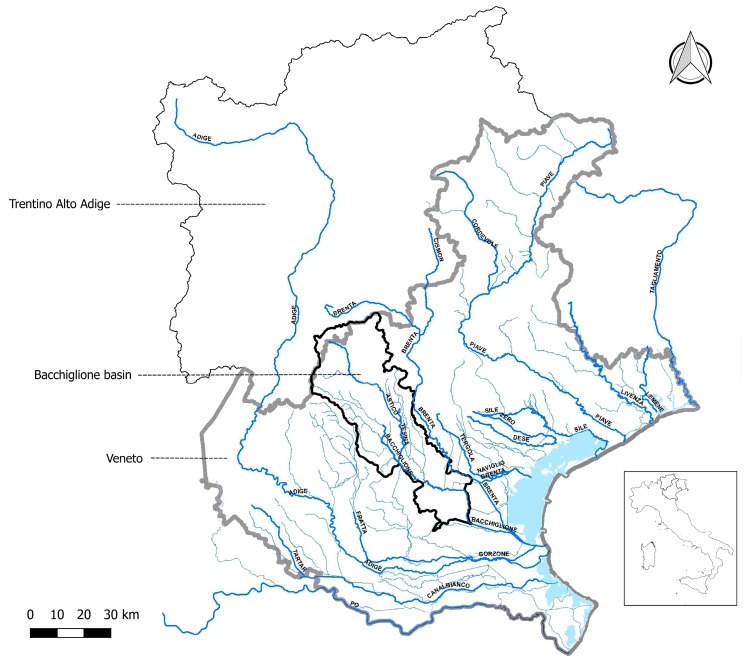
The Bacchiglione basin and neighbouring basins.

**Figure 2:**
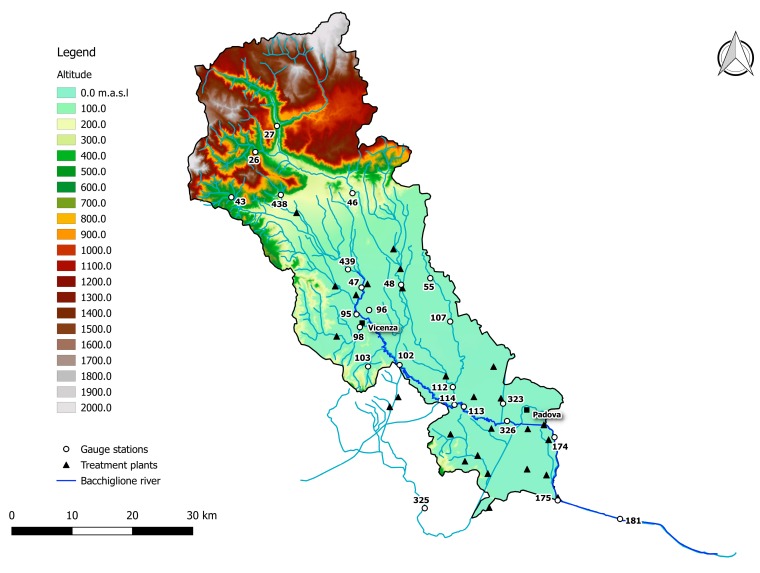
The Bacchiglione basin with gauge stations and treatment plants.

The Bacchiglione basin is located in north-east Italy and appears as a complex hydrographic system, made of two sub-basins with distinct morphological and geo-tectonic characteristics: the Astico–Tesina to the east and the Leogra to the west (Figure 1). It has a maximum altitude of 2.334 m asl and an extension of 1.177 km^2^ (Figure 2). The river Bacchiglione has a length of 118 km and crosses two major cities, Padova and Vicenza, flowing into the Adriatic Sea. It borders to the south-west with the tributary reservoir of the Agno–Guà, to the west with that of the Adige and to the north-east with that of the Brenta. It is characterised by a seasonal variation of the flows: generally high levels in winter and low levels in summer.

In Figure 2, only the treatment plants with a potential of more than 2000 equivalent inhabitants (EI) were reported, because they release more pollution loads and water flow. In the same figure, the white circles show the gauge stations managed by the Regional Environmental Prevention and Protection Agency of Veneto (ARPAV), which measure the flow depth and the physical–chemical parameters: DO%, BOD5, COD, NH4-N, NO3-N, P and *Escherichia (E.) coli*.

**Figure 3:**
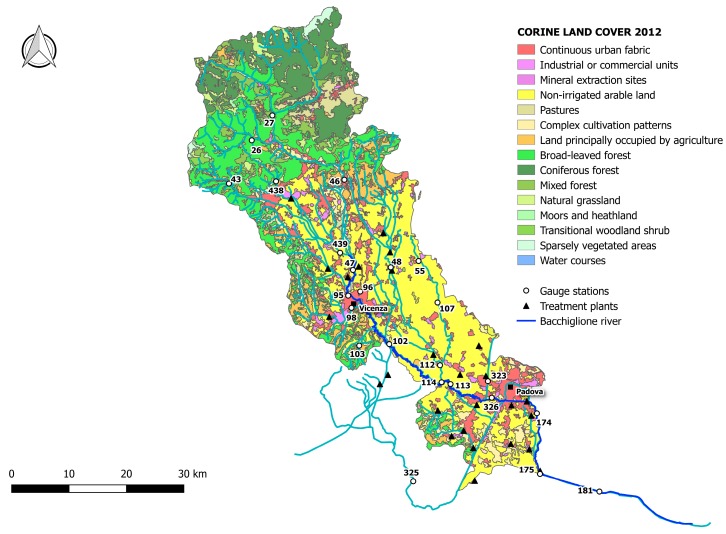
Land use in the Bacchiglione basin.

Mapping the Bacchiglione basin (Figure 3) according to the project Corine Land Cover (CLC) 2012, the mountain area is covered by broad-leaved and coniferous forest by 11.0% and 6.4%, respectively, while the remaining area up to the mouth, including Vicenza and Padova, is constituted by 63.8% of non-irrigated arable land with small spots of discontinuous urban fabric (4.3%). Table 1 reports the percentages of land use in the whole basin.

## 3. Materials and Methods

One of the major causes of river pollution in the Bacchiglione basin is the organic, nutrient, and microbiological load produced by the population and not appropriately treated or eluded by the treatment plants.

This section initially describes the features of the existing WWTPs within the investigated basin and, later, the different analytical steps of the integrated methodology, implemented mainly to investigate the performance of the plants and their correct localisation.

### 3.1. Wastewater Treatment Plant Features

A human presence produces organic, nutrient, and microbiological loads. When these are discharged into water bodies, they determine oxygen consumption and eutrophication phenomena, carrying pathogens dangerous for human health. Other loads could originate in pharmaceuticals or household chemicals like paints, solvents, detergents, pesticides and similar products.

Beside the self-cleaning capacity of the river from the point and diffuse pressure sources, the achievement of water-quality objectives in the receiving body depends on the efficiency of the WWTPs.

To reduce the organic, nutrient, and microbiological loads, biological-treatment systems are normally adopted [36]. Generally, the WWTPs are designed with primary (when appropriate) and secondary (activated sludge aerobic reactor) sections. These plants are able to guarantee an abatement efficiency of 60–95% of organic load, 30–40% of nutrient load [36,37], and at least two logs of 50–60% of microbiological load (through indicator species) [38,39]. To dispose of nutrient loads satisfactorily, these systems are upgraded with a tertiary treatment section. These plants can abate organic pollution as well as nutrients (nitrogen and phosphorus compounds) but cannot reduce substances that are toxic for microorganisms. Therefore, they are designed to reduce only the loads responsible for: (1) oxygen depletion in the water body due to organic load and to the consequent biological activity of heterotrophic microorganisms; (2) algal blooms in closed water bodies (reservoirs, lakes, estuarine zones, lagoons, coastal zones) due to nutrient enrichment processes; (3) dangerous consequences of pathogens due to human use of water. To dispose of almost all the microbiological loads, instead, a disinfection section is necessary. In fact, efficient disinfection systems allow a microbiological abatement of 99.99%, which can give an acceptable guarantee against infections [40]. However, from the environmental point of view, the microbiological load does not appear so important unless related to human use (drinking water production, irrigation, bathing). Moreover, disinfection (with different technological solutions: chlorine compounds, peracetic acid, performic acid, ozone, ultraviolet (UV) rays, ultra-filtration, etc.) can produce dangerous by-products [41], which can have toxic effects on the aquatic ecosystem and can bioaccumulate in the food chain [42].

The 65% of the existing plants within the Bacchiglione basin have a potentiality of more than 10,000 EI and, thus, are also equipped with a tertiary treatment section, while the remaining part has a secondary section.

### 3.2. Integrated Methodology

#### 3.2.1. Mathematical Model

The mathematical model proposed to study the integrity or vulnerability of the river Bacchiglione in terms of the pollution phenomenon is based on the theory of influence diagrams [27,28,29].

The first to introduce the influence diagram to model decision problems with uncertainty were Howard and Matheson 1984 [27]. Problem solving originally displayed the influence diagram in the shape of a decision tree. Subsequently, Shachter 1988 [28] described a way to solve the influence diagram directly without transforming it into a decision tree. The method works through the node-removal and arc-reversal operations, reaching a final diagram with a single utility node, which is that of the optimal decision. In 1992, Shenoy [29] defined another approach to solve the influence diagram, converting it to a valuation network. Shenoy’s algorithm, which has a system of valuations, is slightly more efficient than Shachter’s, which instead has a system of conditional probability functions (in addition to the utility functions).

**Figure 4:**
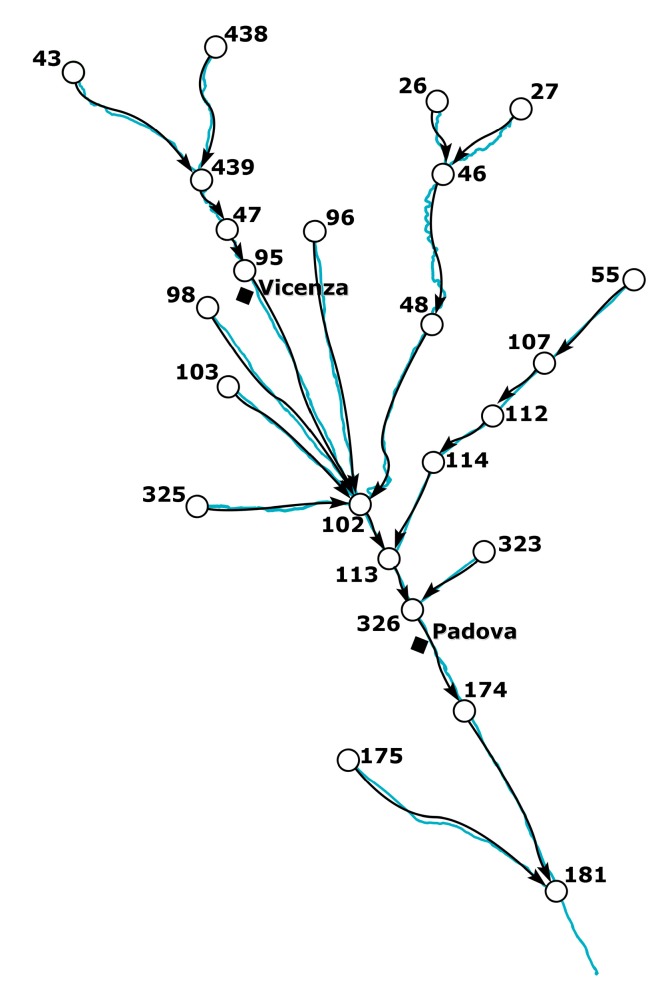
Graphical representation of the river Bacchiglione.

The influence diagram used in the present work is considered only at topological level, without involving the functional and numerical levels dealt with in previous studies. In fact, it is a simple graphical representation whose elements are the nodes, and the relationships between them are illustrated by arrows. The nodes symbolise the cross-sections of the river, where the gauge stations are located, while the arrows, representing the relationships between the functionalities of the different entities, depict the fluvial reaches linking the nodes (Figure 4). This influence diagram at a topological level does not need a probabilistic basis.

The main target of this work is the analysis of the ability of the water body, within its surrounding territorial system, to react to both direct and indirect stresses. The stresses to which each node (entity) is subjected must be identified and quantified. In this study, the stresses are represented by the wastewater flow coming from treatment plants. For each node, *i*, of the graph it is thus possible to associate a vector stress, ${\bar{\xi }}_{i}^{k}$, referring to a specific hazard, *k*, which is related to the physical condition of the integrity of the node, *y_i_*, through the vulnerability function, $f_{i}^{k}$:

(1) $$y_{i}=f_{i}^{k}\cdot ({\bar{\xi }}_{i}^{k}),$$

This function provides a quantitative evaluation of the level of vulnerability or integrity that can be affected by a given stress caused by a natural hazard, *k*. Here, the stress being ${\bar{\xi }}_{i}^{k}$, a scalar quantity, it can be described with a specific index, and the vulnerability function as a simply non–increasing monotonic function, included in the co–domain 0–1:

(2) $$y_{i}=a\left(\frac{e^{-\alpha I_{i}{}^{2}}}{1+e^{-\alpha I_{i}{}^{2}}}\right),$$

where the parameters *a* and *α* define the shape of the function expressed by Equation (2). However, the vulnerability or integrity of a territorial entity cannot depend only on the functionality of the node itself, but also on the functionalities of the other elements. Therefore, the level of functionality of each node on the graph depends on the intrinsic levels of physical integrity of the *i*-th node, *y_i_*, as well as on the of other nodes, ${\bar{x}}_{j}$, related to the *i*-th node:

(3) $${\bar{x}}_{i}={\bar{\phi }}_{i}\left(y_{i},{\bar{x}}_{j},j\in P\left(i\right)\right),$$

where *P(i)* is the set of predecessors of node *i*, that is the set of nodes *j* for which the oriented link (*j*, *i*) exists. In Equation (3) the term ${\bar{x}}_{j}$ can be defined through a scalar monotonic non–decreasing function, with domain and co–domain belonging to the range 0–1 [43]:

(4) $$w_{ij}\left(x_{j}\right)=\left(1-0.1\alpha_{ij}\right)\frac{{\left(1-e^{-\alpha_{ij}x_{j}^{2}}\right)}^{}}{{\left(1-e^{-\alpha_{ij}}\right)}^{}}+0.1\alpha_{ij},$$

where *α_ij_* is a parameter characterising the link (*i*, *j*) and has been calculated in relation to the existent correlations between the entities *i* and *j*.

The functional integrity, ${\bar{x}}_{i}$ varies from 0 to 1, where 1 indicates complete functional integrity and 0 that the entity is not functional. When analysing territorial problems affecting the risk of the functionality loss, like the pollution phenomenon, it is reasonable to optimise the vector function, ${\bar{\varphi }}_{i}$, by considering the conditions of minimum system functionality and maximum risk for the territory. Equation (3) can, thus, be presented as follows:

(5) $$x_{i}=\min{\left(y_{i},w_{ij}\left(x_{j}\right),j:e_{j}\in P\left(i\right)\right)}^{},$$

Therefore, the maximum vulnerability is reached by the territorial system when a condition of minimum functionality occurs for each node [31].

#### 3.2.2. Pressure Sources Indices

The indices used to calculate the physical integrity level had previously been discussed in [31] and are the Non-Treated Loads index and the Total Pollutant Reduction index.

The Non-Treated Loads index measures those loads not provided by treatment plants and that reach the water body without any reduction. It is defined as:

(6) $$I_{NTL,i}=1-\frac{\sum_{r=1}^{n}Q_{tr.p.,r}\cdot c_{tr.p.,r}}{Q_{i}\cdot c_{i}},$$

where *Q_tr.p.,r_* and *c_tr.p.,r_* are, respectively, the flow rate and the concentration of physical–chemical parameters (BOD_5_, COD, NH_4_-N, NO_3_-N, P, and *E. coli*) discharged by n treatment plants located upstream of the node, *i*, in the graph. *Q_i_* and *c_i_* are, respectively, the flow rate and the concentration of physical–chemical parameters measured by the gauge station representing the node, *i*. The index was not calculated for the DO because it is not a direct measure of the real impact of urban wastewater discharges (treated or non-treated), unlike the other parameters. In fact, it appears in an indirect form influenced by the bacterial activity, re-oxygenation process, and temperature of the receiving body. For the treatment plants, the project flow rate was used and it was considered constant for the whole period of study (2007–2015). The flow discharge in each gauge station was calculated, instead, using expeditive mathematical models based on entropy theory [44,45,46,47,48].

A high index value means that some diffused loads may have not been treated at all or, rather, that they could have eluded the treatment plant; while a low value indicates a dilution effect as well as a self-depuration effect provided by the river itself.

The Total Pollutant Reduction index allows evaluating the overall river response to the pollutant load and it is defined as:

(7) $$I_{RED,i}=\frac{Q_{i}\cdot c_{i}}{P_{i}\cdot L},$$

where *Q_i_* and *c_i_* represent, respectively, the flow-rate and the concentration of physical–chemical parameters (COD, BOD_5_, NH_4_-N, and P) measured at the node, *i*, located downstream of the considered river reach, while *L* is the theoretical load of physical–chemical parameters (COD, BOD_5_, NH_4_-N, and P) provided by each single equivalent inhabitant, and *P_i_* is the population of the sub-basin flowing into the node. The index was not calculated for the parameters NO_3_-N and *E. coli* because the theoretical load in the first case is negligible and in the second case is difficult to evaluate. A high index value corresponds here to a low self-protecting capacity of the water body from anthropic pollution loads acting on the specific sub-basin and/or the presence of other pollution sources, while a low index value represents the dilution and self-depuration effect provided by the river itself and/or the efficiency of existing treatment plants.

The theoretical loads of physical–chemical parameters are reported in Table 2 and the population of the sub-basin flowing into the node was estimated according to the 2007–2015 census by the National Institute for Statistics (ISTAT).

#### 3.2.3. Integrity Scenarios

**Figure 5:**
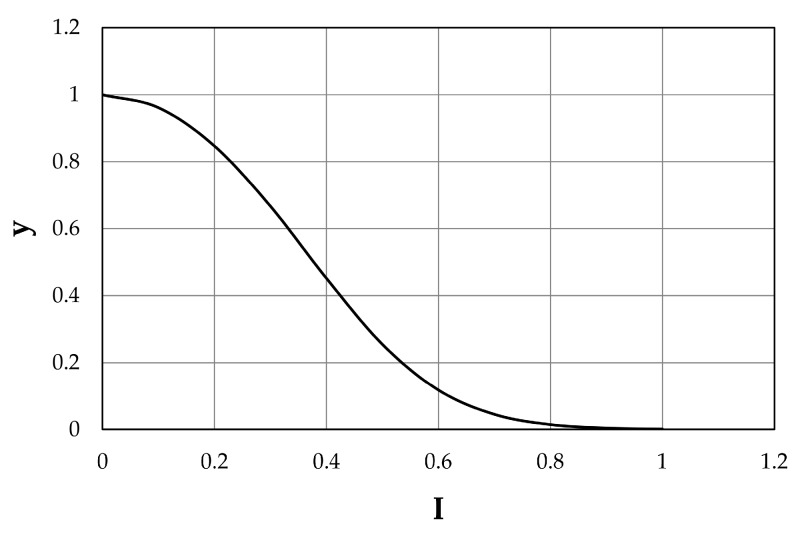
Physical integrity for both indices.

In order to evaluate the integrity or vulnerability scenarios in the Bacchiglione basin, the first step is the determination of the physical integrity level, *y_i_*, by Equation (2) for each index built on the physical–chemical parameters, both of LIM and LIMeco. Such a level was calculated by formulating the hypothesis that three integrity classes (0–0.4; 0.4–0.8; 0.8–1) have to be considered (low, mean, and high), according to a previous work by the authors [14]. In particular, following a thorough analysis of the indices’ distribution, the outer thresholds (0, 1) were picked to represent the limit values of the computed indices and allowed determination of the parameters *a* and *α*, defining thus the shape of the function expressed by Equation (2), reported in Figure 5. The inner thresholds (0.4, 0.8), instead, were chosen considering a condition of environmental caution, making the majority of the two indices’ values fall within the classes of high and mean integrity, as shown in Figure 5. Later, in order to estimate the function *w_ij_(x_j_)*, representing a link between the nodes, a potential functional influence of node *i* on node *j* was investigated. According to different correlations between the index values of the nodes (weak, medium-strong, and strong), three levels of influence were assessed, which allowed estimation of the parameters in Equation (4). In detail, the values 8, 6, and 2 were assigned to each level, respectively. It is important to underline that a gauge station located downstream will be influenced by one upstream of the same reach, but the opposite is not possible.

Finally, two functional integrities, *x_i_*, for each investigated year were assessed according to Equation (5). The first considers the minimum value among all *y_i_* and *w_ij_(x_j_)* obtained for the physical–chemical parameters of the LIM classification until 2010. The second is, instead, the minimum value among all *y_i_* and *w_ij_(x_j_)* obtained for the physical–chemical parameters of the LIMeco classification after 2010. Therefore, for each index and for each year, it is possible to evaluate a unique integrity scenario representing the minimum critical status for the system. Table 3 summarises the main symbols used in the applied methodology.

## 4. Results and Discussion

The proposed integrated methodology was implemented within the river basin according to the integrity scenarios built on the two indices described in Equations (6) and (7) and on the estimated water-quality status, based on the LIM (before 2010) and the LIMeco macro-descriptor (after 2010). In particular, eight cases can occur.

When the water quality of the fluvial reach is lower than “good ecological status”:

- if both the values of functional integrity for the Non-Treated Loads and the Total Pollutant Reduction indices are low, the treatment plants which discharge in the water body might not work properly or might not be adequately sized to dispose of the load produced by the population;
- if the functional integrity is high for *I_NTL_* and low for *I_RED_*, the area could present uncontrolled discharge points which reach the water body without undergoing purification by the treatment plants;
- if the functional integrity is low for *I_NTL_* and high for *I_RED_*, the treatment plants might not be able to reduce the pollution load according to the provisions of the WFD, and the deterioration of the river could also be due to the presence of other sources of pollution, such as those coming from agricultural and industrial activities;
- if the functional integrity for both indices is high, the water body might be subject to other sources of pressure.

When the water quality of the fluvial reach is equal to or greater than “good ecological status”:

- if the functional integrity for the Non-Treated Loads and the Total Pollutant Reduction indices is low, the river might have managed to completely dispose of the pollution load coming from upstream. The presence of unsuitable and undersized treatment plants could change the water quality during the summer months, due to the low flow rate or the absence of turbulent currents;
- if the functional integrity is high for *I_NTL_* and low for *I_RED_*, the fluvial reach shows a high self-purification capacity. The presence of uncontrolled discharge points could represent the cause of deterioration, both in the case of low flow rate and in modified hydrodynamic conditions of the current;
- if the functional integrity is low for *I_NTL_* and high for *I_RED_*, the presence of inefficient plants and other sources of pollution spread on the sub-basin could threaten the water quality;
- if the functional integrity for both indices is high, the area might not contain sources of pollution.

Table 4 summarises all the possible cases.

In the first four cases, the low quality of the river underlines the loss of functionality for the water body (condition of damage). In cases 5, 6 and 7 even if the river has managed to react to the point and diffuse pressure sources on the whole basin, the high and mean vulnerability scenarios highlight a condition of potential damage. In the last case, the territory faces no pollution risk.

According to the case (real or potential damage), the municipal decision-maker can choose the type of intervention to be adopted, not only to mitigate the effects caused by the pollution source, but also to reduce the risk of the degradation phenomenon. These interventions can be of a structural and non-structural nature. The first are works carried out in the whole river basin or in small portions of the territory. They may include the implementation of new treatment plants and sewage systems, the upgrade of existing ones, the construction of works in the riverbed to increase the turbulence phenomena and, thus, the diffusion and dilution of pollutant loads and the re-naturalisation of the fluvial reaches to increase their self-purification capacity. The second cover: the strengthening of water bodies and river-basin monitoring; the control of discharge points; the adoption of safeguarding and restriction measures; correct urban planning and land use; the definition of good agricultural practices; territory maintenance and cleaning actions; research into advanced technologies for the treatment of wastewater; and the relocation of the urban and industrial settlements most at risk. 

Most of the time, the difficulties encountered in modifying habits and behaviors direct the choice towards structural interventions, even if financially disadvantageous. However, thanks also to the commitment of the United Nations, the concept of resilience has recently been retrieved, indicating a system’s capacity, when exposed to risks, to resist, absorb, adapt to and recover from the effects of a danger in a timely and efficient manner, even through the conservation and restoration of its basic essential structures and functions. The first scholar to introduce the concept of resilience in the ecological sciences was Holling [49], stating that “it is a measure of the persistence of systems and of their ability to absorb change and disturbance and still maintain the same relationships between populations or state variables” [50]. Resilience allows a new approach to the choice of action typology to be adopted based on a holistic and farsighted cost/benefit analysis. In this way, the municipal decision-maker moves from the comprehension of a phenomenon, goes through the occurrence potentiality evaluation of future degradation processes, weighs costs and benefits of different solutions, and reaches the adoption of the most appropriate mitigation measures. These steps are necessary for the correct planning and design of interventions within the basin and a more balanced choice of structural/non-structural actions. In view of this, the solutions for risk adaptation start to have an important role compared to more invasive and expensive structural works. In particular, cases 1, 2, and 3 suggest more short-term structural interventions, while case 4 leads to medium-term non-structural and case 5 to medium-term structural interventions. The other two cases, 6 and 7, imply long-term non-structural actions, while the last case implies none (Table 5).

**Figure 6:**
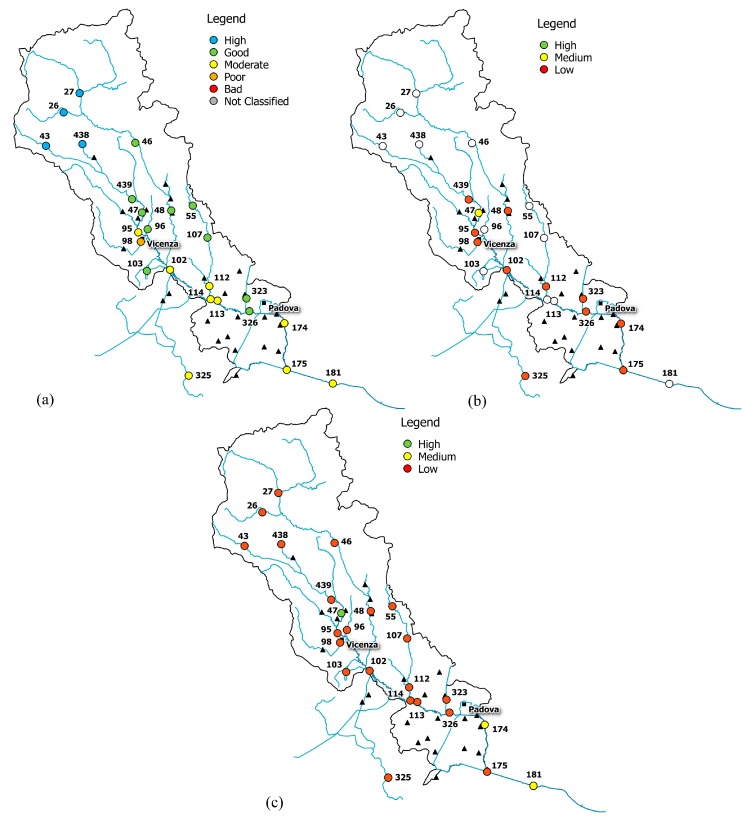
(**a**) Water-quality classification according to the macro-descriptor LIM; (**b**) level of functional integrity for the Non-Treated Loads index; (**c**) level of functional integrity for the Total Pollutant Reduction index (year 2008).

**Figure 7:**
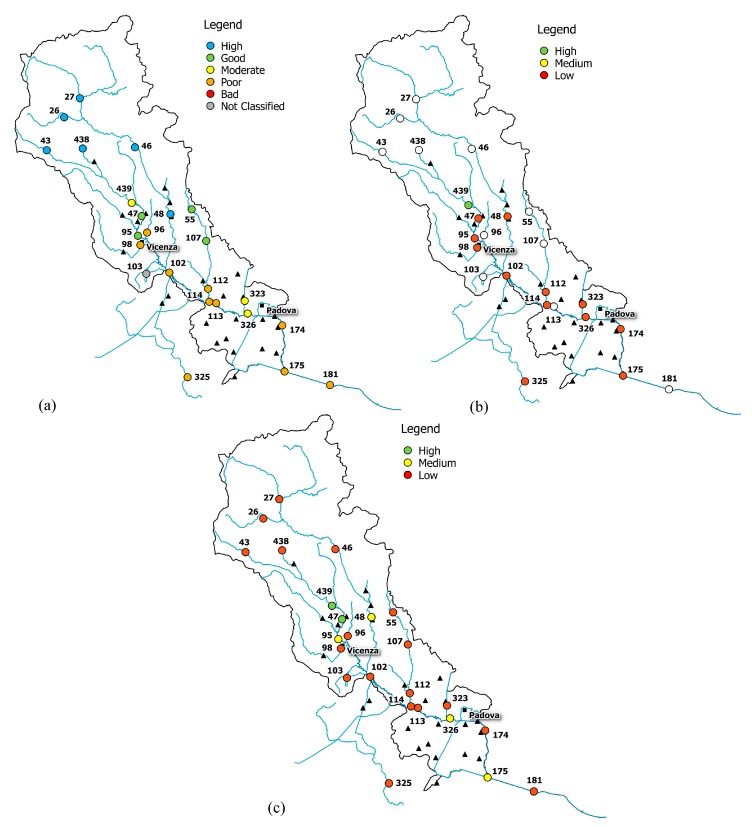
(**a**) Water-quality classification according to the macro-descriptor LIMeco; (**b**) level of functional integrity for the Non-Treated Loads index; (**c**) level of functional integrity for the Total Pollutant Reduction index (year 2012).

**Figure 8:**
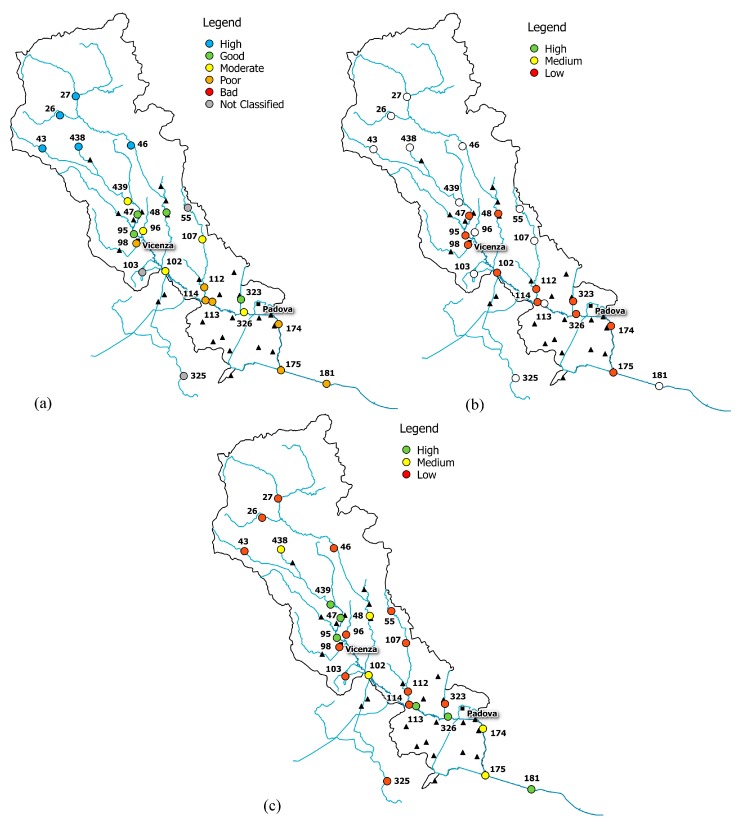
(**a**) Water-quality classification according to the macro-descriptor LIMeco; (**b**) level of functional integrity for the Non-Treated Loads index; (**c**) level of functional integrity for the Total Pollutant Reduction index (year 2015).

The combined reading of integrity scenario, water-quality status, information on basin morphology and land use allowed the identification of some conditions of real and potential damage for the Bacchiglione, reported in Figure 6, Figure 7 and Figure 8.

The upper part of the basin (gauge stations n. 26, 27, 43, 46, and 438) shows low functional integrity for the Non-Treated Loads index throughout the reference period (Figure 6, Figure 7 and Figure 8). For these nodes, instead, the Total Pollutant Reduction index was not calculated due to the absence of treatment plants. Such results highlight a high pollution load produced by the population and discharged directly in the receiving body without undergoing any treatment. Despite this outcome, the water-quality status ranges between “high” and “good” throughout the investigated period (2007–2015), probably thanks to a high self-purification capacity of the river. The latter is improved by turbulence phenomena due to steep reaches (Figure 2) and naturalised fluvial stretches, covered by broad-leaved and coniferous forest (Figure 3). Since this area falls in case 5, the territory action planning should focus more on medium-term structural interventions (Table 5). In fact, the presence of significant civil loads produced by small municipalities spread over the mountain, which might threaten the water quality, especially in the dry periods, should lead decision-makers to design new WWTPs and plan a series of actions aimed at increasing the river’s resilience.

In the mid-valley basin (gauge stations n. 439, 47, and 48), between 2007 and 2010, the value of functional integrity for the Non-Treated Loads and the Total Pollutant Reduction indices is low (Figure 6). This shows that some civil loads might not be appropriately disposed of by the WWTPs, while others discharge directly in the receiving body. After 2010, the situation changes and the area depicts a high integrity condition for the Total Pollutant Reduction index (Figure 7 and Figure 8). This underlines the fact that the civil load does not represent the main threat for the river any longer. The “high” or “good” quality status throughout the period (2007–2015) demonstrates not only the water’s resilience to the pollution phenomenon, but also the positive effects deriving from continuous monitoring activities and the building of new WWTPs after 2010, following the WFD adoption. In detail, structural interventions in the area allowed a transition from a high-vulnerability condition (case 5) to a medium one (case 7). However, the low functional integrity for the Non-Treated Loads index could lead to great river degradation phenomena over the years. Facing a condition of potential damage (case 7), the municipal decision-maker should perform a series of long-term non-structural actions able to improve the existing plants’ treatment technologies and increase the self-purification capacity of the water body. For example, the re-naturalisation of parks and green areas and the funding of research on new wastewater-treatment technologies might boost socio-economic development of the territory. Besides, the threat of fluvial degradation due to the presence of arable land (Figure 3), which discharges pollutants into the receiving body following the washout, should lead the decision-maker to adopt good land use practices and introduce new cultivation and ploughing techniques.

The remaining part of the basin, transiting Vicenza and Padova, shows low functional integrity for both indices, depicting a high-vulnerability scenario. Only in the last few years, near the city of Padova, has the Total Pollutant Reduction index gone from low to medium and high values, representing how the load produced by the population is not the unique pollution source. “Good” or “moderate” water quality (Figure 6), instead, becomes “moderate” or “poor”, respectively (Figure 7 and Figure 8), due to the presence of the two big urban centres and industrial areas that stress the river. Land use and cover, being quite homogeneous in this area (Figure 3), are not significant sources of pollution. The absence of point discharges along the tributaries demonstrates, instead, “good” water quality. Considering the real damage condition (case 1), the municipal decision-maker must intervene promptly with structural actions able to reduce the point and diffuse pressure sources and increase the resilience of the river and surroundings. The choice of the appropriate mitigation measure must be anticipated by a careful evaluation of the technical and economic feasibility of the interventions.

## 5. Conclusions

The methodology proposed in this work represents a useful tool to help municipal decision-makers plan and design short-, medium- and long-term interventions and actions in the river and its surroundings, in order to preserve or reach “good ecological status”, as predicted by the Water Framework Directive 2000/60/EC and Italian laws. This combined approach could contribute to drawing greater attention towards the employment of more efficient decision support systems (DSS) for the achievement of the WFD goals. In particular, it would be interesting to implement technical guidelines to support the directive, which would be able to inform the water authorities and managers on the pollution risk in strongly anthropised, industrial, and agricultural areas, and on how the water body resists external pressures. In addition, it would assist decision-makers in the choice of the best mitigation action or intervention according to the real or potential damage condition. 

The present methodology was adopted by the same authors in a previous study, but it was extended here to a larger monitoring data set (nearly 10 years) and a wider range of chemical and physical parameters, comparing the old classification with the new Italian monitoring system (decree n. 260/2010), in compliance with the WFD.

The procedure, applied to the Bacchiglione, a sample river basin in northern Italy, takes into account both the water-quality status and the behaviour of the system under stress, evaluated through a mathematical integrity model. In particular, the latter was implemented according to the Non-Treated Loads index, which considers the influence of the diffused loads, untreated or eluded by the treatment plant, and to the Total Pollutant Reduction index, which estimates the self-protection action of the water body against anthropic loads acting on the basin. By combining the integrity scenario, water-quality status, information on basin morphology and land use, the different conditions of real and potential damage for the Bacchiglione were identified.

The mountain area of the basin shows a high civil load produced by small municipalities, which could represent a threat for the river especially at minimum values of water discharge. The condition of pollution risk should thus lead the decision-maker to plan medium-term structural actions focused on designing new WWTPs and increasing the river’s resilience.

The mid-valley basin underlines both the high resilience of the water body and the positive effects of continuous monitoring activities and of new WWTPs built after 2010, thanks to the adoption of the WFD. Nevertheless, even in this case the presence of high-vulnerability scenarios directs the decision-maker towards a series of long-term non-structural interventions, such as research funding, improving existing plants’ treatment technologies, re-naturalisation of fluvial reaches through parks and green areas, the adoption of good practices for arable land use, and the introduction of new, lower-impact cultivation and ploughing techniques.

The remaining part of the basin transiting Vicenza and Padova highlights a recent deterioration of water quality, mainly due to the presence of the two big urban centres and industrial areas that stress the river. The condition of real damage pushes the decision-maker to intervene promptly with structural actions able to reduce the point and diffuse pressure sources and increase the resilience of the river and its surroundings.

## Figures and Tables

**Table 1:** Classes of land use in the Bacchiglione basin according to Corine Land Cover 2012.

Type of Land Use	%
Discontinuous urban fabric	4.3
Industrial or commercial units	1.1
Mineral extraction sites	0.1
Non-irrigated arable land	63.8
Pastures	1.9
Complex cultivation patterns	2.8
Agriculture + natural veg.	3.3
Broad-leaved forest	11.0
Coniferous forest	6.4
Mixed forest	2.8
Natural grassland	1.1
Moors and heathland	0.4
Transitional woodland-scrub	0.5
Sparsely vegetated areas	0.3
Water courses	0.2

**Table 2:** Theoretical loads of physical–chemical parameters.

Theoretical Load	Value (Grams per inh., per Day)
COD	100
BOD_5_	60
NH_4_-N	7
P	2

**Table 3:** List of main symbols.

Symbol	Description
${\bar{\xi }}_{i}^{k}$	vector stress referred to natural hazard of the *i*-th node
*k*	natural hazard
$f_{i}^{k}$	vulnerability function of the *i*-th node
*y_i_*	level of physical integrity of the *i*-th node
*a, α*	coefficients of the level of physical integrity
${\bar{x}}_{j}$	vector level of functionality of the *j*-th node linked to the *i*-th node
*w_ij_(x_j_)*	scalar level of functionality of the *j*-th node linked to the *i*-th node
*α_ij_*	coefficient of the level of functionality
${\bar{x}}_{i}$	vector functional integrity of the *i*-th node
*x_i_*	scalar functional integrity of the *i*-th node
${\bar{\varphi }}_{i}$	vector function of optimisation of the *i*-th node
*P(i)*	set of predecessors of the *i*-th node
*I_NTL_*	Non-Treated Loads index
*I_RED_*	Total Pollutant Reduction index
*Q_tr.p._*	flow rate discharged by treatment plant
*c_tr.p._*	concentration of physical–chemical parameter discharged by treatment plant
*Q*	flow rate measured by gauge station
*c*	concentration of physical–chemical parameter measured by gauge station
*L*	theoretical load of physical–chemical parameter
*P_i_*	population of basin flowing into the *i*-th node

**Table 4:** The eight possible cases occurring in the river basin.

Case	Ecological Status	*I_NTL_*	*I_RED_*	Integrity	Damage
1	<Good	Low	Low	Low	Real
2	<Good	Low	High	Mean	Real
3	<Good	High	Low	Mean	Real
4	<Good	High	High	High	Real
5	≥Good	Low	Low	Low	Potential
6	≥Good	Low	High	Mean	Potential
7	≥Good	High	Low	Mean	Potential
8	≥Good	High	High	High	Potential

**Table 5:** Intervention/action typology according to resilience and integrity scenario.

Case	Resilience	Integrity	Damage	Intervention/Action	Timing
1	Low	Low	Real	Structural > Non-Structural	Short-term
2	Low	Mean	Real	Structural ≥ Non-Structural	Short-term
3	Low	Mean	Real	Structural ≥ Non-Structural	Short-term
4	Low	High	Real	Structural ≤ Non-Structural	Medium-term
5	High	Low	Potential	Structural ≥ Non-Structural	Medium-term
6	High	Mean	Potential	Structural ≤ Non-Structural	Long-term
7	High	Mean	Potential	Structural ≤ Non-Structural	Long-term
8	High	High	Potential	None	Long-term

## Appendix A

The macro-descriptor used to evaluate the ecological status of a surface water body was LIM until 2010 [11]. It was defined as the sum of the scores associated to seven physical–chemical parameters (DO%, BOD5, COD, NH4-N, NO3-N, P, and *E. coli*), according to Table A1. The score of the LIM was successively combined with the score obtained by the extended biotic index (EBI) [51], which was a synthetic index of the biological quality of the river. Higher values of the EBI corresponded to better water quality. The ecological status of the water body was thus expressed in terms of five quality classes, which varied from 1 (best) to 5 (worst); it was attributed to the river cross-section or reach, taking into consideration the worst result among those obtained between the EBI and the macro-descriptor LIM (Table A2). Finally, the attribution of the quality to the water body occurred by comparing the data of the ecological status to those of the main chemical micropollutants (e.g., metals, organochlorine compounds and phytopharms), as indicated in Table A3. Water quality could vary from high to bad, according to the five classes.

**Table A1:** Different pollution levels expressed by the macro-descriptor LIM. The reported levels correspond to concentration or percentage and are directly measured after sampling.

Parameter	Level 1	Level 2	Level 3	Level 4	Level 5
100-OD (% sat.)	≤10	≤20	≤30	≤50	>50
BOD_5_ (O_2_ mg/L)	<2.5	≤4	≤8	≤15	>15
COD (O_2_ mg/L)	<5	≤10	≤15	≤25	>25
NH_4_ (N mg/L)	<0.03	≤0.10	≤0.50	≤1.50	>1.50
NO_3_ (N mg/L)	<0.3	≤1.5	≤5.0	≤10.0	>10.0
Total phosphorous (P mg/L)	<0.07	≤0.15	≤0.30	≤0.60	>0.60
*Escherichia coli* (UFC/100/mL)	<100	≤1.000	≤5.000	≤20.000	>20.000
Score to be attributed for each analysed parameter (75° percentile of the monitoring period)	80	40	20	10	5
Pollution level expressed by the LIM	480–560	240–475	120–235	60–115	<60

**Table A2:** Determination of ecological status by the extended biotic index (EBI) and LIM.

Parameter	Class 1	Class 2	Class 3	Class 4	Class 5
EBI	≥10	8–9	6–7	4–5	1, 2, 3
Pollution Level expressed by macro-descriptor LIM	480–560	240–475	120–235	60–115	<60

**Table A3:** Determination of environmental status according to the sulphur-emission control area (SECA) class and the concentration of additional parameters.

Ecological Status (SECA) ⇒	Class 1	Class 2	Class 3	Class 4	Class 5
Pollutant concentration ⇓					
≤threshold value	High	Good	Moderete	Poor	Bad
>threshold value	Poor	Poor	Poor	Poor	Bad

After 2010, ecological status was evaluated through the macro-descriptor LIMeco. It is attributed to the river cross-section or reach for each year as a weighted average of the LIMeco for each sampling. The latter is estimated as the average value of the scores associated with the concentration of each parameter according to Table A4 [26].

**Table A4:** Thresholds for score attribution to each parameter.

Parameter		Level 1	Level 2	Level 3	Level 4	Level 5
	Score *	1	0.5	0.25	0.125	0
100-OD (% sat.)	Thresholds	≤|10|	≤|20|	≤|40|	≤|80|	>|80|
NH_4_ (N mg/L)		<0.03	≤0.06	≤0.12	≤0.24	>0.24
NO_3_ (N mg/L)		<0.6	≤1.2	≤2.4	≤4.8	>4.8
Total Phosphorous (P mg/L)		<50	≤100	≤ 200	≤400	>400

* Score attributed to the single parameter.

The attribution of quality to the water body is based on the limits of Table A5 [26]. The water quality, expressed in five classes, can vary from high to bad.

**Table A5:** Water-quality classification according to the LIMeco limits.

Status	LIMeco
High	≥0.66
Good	≥0.50
Moderate	≥0.33
Moderate	≥0,17
Bad	<0.17
